# Balanced Propofol Sedation in Patients Undergoing EUS-FNA: A Pilot Study to Assess Feasibility and Safety

**DOI:** 10.1155/2011/542159

**Published:** 2011-07-12

**Authors:** N. Pagano, M. Arosio, F. Romeo, G. Rando, G. Del Conte, A. Carlino, G. Strangio, E. Vitetta, A. Malesci, A. Repici

**Affiliations:** ^1^Dipartimento di Gastroenterologia, Istituto Clinico Humanitas, Via Manzoni 56, MI, 20089 Rozzano, Milano, Italy; ^2^Dipartimento di Anestesiologia, Istituto Clinico Humanitas, Via Manzoni 56, MI, 20089 Rozzano, Milano, Italy

## Abstract

*Introduction and aims*. Balanced propofol sedation (BPS) administered by gastroenterologists has gained popularity in endoscopic procedures. Few studies exist about the safety of this approach during endosonography with fine needle aspiration (EUS-FNA). We assessed the safety of BPS in EUS-FNA. *Materials and methods*. 112 consecutive patients, referred to our unit to perform EUS-FNA, from February 2008 to December 2009, were sedated with BPS. A second gastroenterologist administered the drugs and monitorized the patient. *Results*. All the 112 patients (62 males, mean age 58.35) completed the examination. The mean dose of midazolam and propofol was, respectively, of 2.1 mg (range 1–4 mg) and 350 mg (range 180–400). All patients received oxygen with a mean flux of 4 liter/minute (range 2–6 liters/minute). The mean recovery time after procedure was 25 minutes (range 18–45 minutes). No major complications related to sedation were registered during all procedures. The oxygen saturation of all patients never reduced to less than 85%. Blood systolic pressure during and after the procedure never reduced to less than 100 mmHg. Conclusions. In our experience BPS administered by non-anaesthesiologists provided safe and successful sedation in patients undergoing EUS-FNA.

## 1. Background

Propofol is a short-acting sedative, agonist of *γ*-aminobutyric acid receptor in the central nervous system [1]. Its use has principally been limited to anesthesiologists for the induction and maintenance of deep sedation in patients undergoing surgical procedures. Recently propofol use has extended to sedation in endoscopic procedure, administered by anesthesiologists or by gastroenterologists [2]. 

Propofol administered by nonanesthesiologists has shown to be safe for an upper endoscopy, a colonoscopy, and advanced endoscopic procedures, such as an ERCP and an EUS [3]. For endoscopic procedures the use of midazolam, with or without meperidine, in combination with propofol is called balanced propofol sedation (BPS). It has been shown that BPS compared to propofol alone decreases total propofol doses required and increases patient comfort [4–6].

## 2. Aim of the Study

The purpose of this preliminary study was to assess safety and feasibility of gastroenterologist-administered BPS for operative upper EUS in a tertiary referral center with a previously established program for colonoscopy sedation with propofol. 

## 3. Patients and Methods

The study received approval from our institutional review board. From February 2008 to December 2009 patients who presented at our unit for an operative EUS were eligible for the study if they were age 18 years or older, American Society of Anesthesiology (ASA) class I or II, and capable to provide written informed consent for study participation. Exclusion criteria were inability to provide informed consent, history of allergic reactions or hypersensitivities to midazolam, propofol, eggs, or soybeans, high-risk head and neck anatomy (Mallampati > 2) that may complicate airway rescue, sleep apnea syndrome, and ASA  class > II. 

Patients underwent BPS administered by a gastroenterologist who was not involved in the endoscopic procedure. The physicians administering sedation were certified in advanced cardiac life support (ACLS) and had successfully completed an intensively structured training program also in the use of laryngeal mask. An anesthesiologist was on call during the procedure. After a single dose of midazolam (0.05 mg/Kg), a starter bolus of 0.5 mg/kg of propofol was administered. Repeated boluses of 10 to 20 mg of propofol were than administered on demand with a 1- to 2-minute interval for the whole time of the procedure. Propofol boluses frequency and dose were titrated on the patient response, including vital signs and manifestations of restlessness or discomfort. The maximum dose allowed to be administered was 400 mg. 

Baseline vital signs (heart rate, blood pressure, and oxygen saturation) were obtained in all patients before induction of sedation. Throughout the procedure, all patients received oxygen 4 L/min by nasal cannula. Continuous pulse oximetry and end-expiratory carbon dioxide (EECO_2_) was monitorized. Blood pressure was measured every 5 minutes. 

The following parameters were recorded: patient demographics, procedure indication and duration, EUS findings, midazolam dose, propofol dose, and number of FNA passes. The baseline values and changes in vital signs or oxygen saturation (SpO_2_) from the baseline were recorded. Complications were recorded, including hypoxia, defined as a reduction in oxygen saturation to less than 85% for more than 20 seconds, use of supplemental oxygen (O_2_) by nasal cannula (NC) in excess of 4 L/min, positive pressure ventilation (PPV) or laringeal mask use, hypotension, bradycardia. EUS was performed by one experienced endosonographer who had practiced for 2 years after completing a therapeutic endoscopy fellowship and performed over 500 EUS procedures yearly. The echoendoscope was inserted once the patient was responsive only to mild prodding and the tongue was flaccid to manual palpation. EUS-guided FNA (EUS-FNA) was carried out in a standard fashion by using a 22-gauge needle. After the procedure, the patients were transported to the recovery room where blood pressure, SpO_2_, and heart rate were measured continuously until discharge. Discharge was possible when Glasgow Coma Scale score of the patient was >9 and OSSA scale was 5 (see Table 2).

## 4. Results

Between February 2008 and December 2009, 253 consecutive patients were considered for enrollment. Among these, 53 patients were excluded because their asa class was III, 34 patients were excluded because their Mallampati score was more than 2, 10 because of a history of sleep apnea syndrome, 44 patients refused to provide informed consent to participate in the study. Overall 112 patients were included in the study. Demographic and baseline characteristics of the patients are shown in Table 1, as well as indications for EUS-FNA. The most common indication was the evaluation of a known or suspected pancreatic mass or cyst. The mean dose of propofol given was 350 mg (range 180–400 mg), and the mean dose of midazolam was 2.1 mg (range 1–4 mg). All patients received supplemental oxygen in nasal cannula at the mean flux of 5 L/min. The mean procedure time in our study was 42.96 ± 15 min. A mean of 3 passes of EUS-FNA was performed (range 2–5). The accuracy of EUS-FNA was 75.3%. One endoscopic complication occurred (0.9%), a perforation of the duodenal bulb in a patient with a pseudodiverticulum of the bulb subsequent to an ulcer. After an attempt of conservative management with the positioning of a nasogastric drainage, total parenteral nutrition and intravenous antibiotic therapy the patient was referred to the surgical unit because of peritonism. The patient is doing well after 3 months of followup. In 5 patients (4.5%) the blood pressure reduced to less than 90 mmHg during an interval of time between two measurements (10 minutes). Administering fluids allowed a rapid increase of the blood pressure above the target value. In 3 patients (2.7%) the heart rate decreased to less than 50 pulses per minute for more than 1 minute. With the administration of intravenous buscopan, the heart rate rose back to more than 50 pulses per minute in few seconds in all these patients. In 5 patients (4.5%) oxygen saturation decreased to less than 90% while they were receiving oxygen with a 4 L/min flux. Two patients rapidly recovered increasing in the flux to 8 L/min, two patients needed mask ventilation for few minutes with complete recovery of a normal blood oxygenation, but this event required the stop of the procedure that was completed with anesthesiological assistance. In one patient (0.9%) the insertion of a laryngeal mask was necessary for a desaturation episode, with an oxygen saturation value of less than 85% lasted more than 3 minutes despite mask ventilation. In this case the procedure was definitively stopped without performing FNA. In all the other patients the blood pressure never decreased under 90 mmHg, the heart rate never reduced to less than 50 pulses per minute and the oxygen saturation never reduced under 85%. Mean postprocedural observation time was 25 min (range 18–45 min).

## 5. Discussion

This study was designed to assess the safety and feasibility of BPS administered by nonanaesthesiologist in EUS-FNA. In our experience this protocol of sedation resulted to be feasible and safe with an adequate training of medical and nursing staff. The guidelines of the American Society of Gastrointestinal Endoscopy (ASGE) do not recommend anesthesiological assistance for routine endoscopy in healthy patients because of excessive costs [7]. Anesthesiological associations have expressed concern about the use of propofol by nonanaesthesiologists because of the possible need for assisted ventilation [8]. A meta-analysis found that the use of propofol by nonanaesthesiologists is not inferior to other agents when used for EGD or ERCP/EUS and, compared to traditional protocols of sedation, appears to lower the risk of cardiopulmonary complications during colonoscopy [9]. In terms of efficacy, recovery, and complications, the use of propofol compared to standard sedation for endoscopy offers a more rapid induction and recovery from sedation and faster discharge postprocedure [10–12]. Several studies have shown that coadministration of benzodiazepine and/or opioids during endoscopic procedures (EGD, colonoscopy, and ERCP), also known as “balanced propofol sedation” (BPS) enhances the sedative effect of propofol and allows titration of propofol to moderate sedation [13]. This regimen also allows total propofol dose to be reduced by about 50% and makes propofol administration smoother by prolonging the interval between doses [14]. Some reports failed to show a reduction in propofol dose with the use of midazolam [15]. Comparing balanced propofol sedation with midazolam alone for an ERCP, propofol is preferred by patients and physicians and shortens recovery time [16, 17]. While its use for EGD and colonoscopy has been extensively reported, there are limited data describing the use of propofol sedation for EUS [18, 19]. 

There are reports on propofol sedation for EUS administered by nonanesthesiologist physicians in boluses or by target-controlled infusion. The safety of Nurses Administered Propofol Sedation (NAPS) and the variables associated with complications from its use for EUS have been also described [20]. In the paper of Fatima et al., by using univariate analysis, patient age, propofol dose, procedure length, and EUS-FNA were not associated with the risk of any sedation-related complications [21].

In our center there has been a strong interest in propofol sedation in the last 4 years, in particular for colonoscopy sedation. Since 2006, our staff has performed more than 5000 colonoscopies with BPS. Nevertheless, features of EUS and, in particular, EUS-FNA procedure features are consistently different from colonoscopy. EUS-FNA is a difficult and time-consuming procedure. Upper endoscopic procedure are at higher risk of respiratory complications as reported in the literature [22]. Beside this, EUS instruments have a major caliber compared to gastroscopes. An EUS-FNA procedure lasts more than a gastroscopy or a colonoscopy and, for safety reasons, during FNA a deeper level of sedation is often required. 

The current study was designed to assess the feasibility and the safety of EUS-FNA performed with BPS sedation administered by gastroenterologists. The administration of propofol by gastroenterologist may be not as cost-effective as when it is administered by trained nurses, but, at present, it is the only way allowed in our institution. During the study, an anaesthesiologist was on call in case of adverse events. The anaesthesiologist was free of other duties attending only to nonclinical activities and ready to reach the endoscopic suite in few minutes.

In our series we found no major complications. All but one procedures were completed. Minor adverse events occurred in 7 patients (6.3%) and were easily treated. Overall, 5 patients (4.5%) had a decline in SBP <90 mm Hg. All episodes of hypotension were treated by increasing the rate of IV fluids. The episodes of hypotension did not require discontinuation of the procedure. The mean procedure time in our study was 42.96 ±15 min, not different from reported data [23]. In the current series, we found a decrease in SpO_2_ < 25% in 5 patients (4.5%), rapidly responsive to supplemental O_2_ administration in 2 patients; 3 patients (2.7%) required assisted ventilation. One procedure (0.9%) was not completed due to prolonged hypoxia treated by laryngeal mask positioning. A single-center, prospective, randomized trial comparing standard sedation versus propofol for ERCP reported no cases of assisted ventilation in the arm of patients receiving propofol [24]. In the studies where propofol was administered by non-anaesthesiologist for outpatient undergoing EUS, no episodes of assisted ventilation were reported [25, 26]. Whether the continuous infusion of propofol lowers the risk of respiratory adverse events compared to boluses administration has never been investigated in the adult population. Experience in the paediatric setting seems to be slightly in favour of fractionated doses administration [27]. 

In our experience BPS can be a safe protocol of sedation for healthy patients undergoing EUS-FNA. Comparing our data to the literature there seems to be a difference in minor complication rate. In particular we experienced the need for mask ventilation in 3 patients, in one case with the use of laryngeal mask. We also had in our series one perforation (0.9%). This observational study was not designed to assess the difference of complication between different protocols of sedation, but comparing data to an historical cohort of patients that underwent EUS-FNA with anesthesiological assistance a similar rate of minor complication was found. In particular a perforation of the duodenal bulb was reported in the cases with anesthesiological assistance. The diagnostic yield of FNA in this series is similar to the literature data. The major concern of propofol sedation is the respiratory depression. The results of our series stress the importance of the training in the airway management of the staff involved in BPS. Laryngeal mask use, in particular, should be considered mandatory for the gastroenterologists administering this kind of sedation. 

In conclusion, our experience suggest a good profile of safety of BPS administered by gastroenterologist in EUS-FNA procedures. According to our results no significant increase in the procedure-related complication rate was registered and the diagnostic yield of FNA performed with this protocol of sedation is not different from the literature data. Gastroenterologist administering propofol for EUS-FNA must be skilled in advanced life support, with particular regard to airway management. Laryngeal mask, having a shorter learning curve than orotracheal intubation, could be the rescue procedure of choice in patients undergoing oxygen desaturation nonresponsive to mask ventilation.

## Figures and Tables

**Table 1:** Baseline population characteristics.

Age, median years (range)	58 (25–86)
Male/female	62/50
ASA grade	
Class I	68
Class II	34
Indication for FNA	
Mediastinal nodes	26
Submucosal lesion	14
Pancreatic mass	72
Needle passes (range)	3 (2–5)
Baseline hemodynamics, median (range)	
Mean blood pressure mmHg	125 (85–168)
Pulse, per minute	71 (48–90)
SaO_2_, %	96 (94–100)

ASA, American Society of Anesthesiologists' grade.

**Table 2:** Observer's scale for sedation and alertness (OSSA) score.

Score	Responsiveness	Eyes
5	Responds readily to name	Clear and no ptosis
4	Lethargic response to name	Glazed or mild ptosis (less than half of eye)
3	Responds only when called loudly or repeatedly	Marked ptosis (more than half of eye)
2	Respond after mild prodding or shaking	
1	Unresponsive to mild prodding or shaking	
